# Pulsatility Assessment of Cerebral Perforating Arteries Using Submillimeter‐Resolution Dual‐VENC Phase‐Contrast MRI at 3T

**DOI:** 10.1002/jmri.70218

**Published:** 2025-12-27

**Authors:** Jianing Tang, Sang Hun Chung, Maria Gamez, Tianrui Zhao, Michael S. Wolf, Dilip K. Pandey, Philip B. Gorelick, Lirong Yan

**Affiliations:** ^1^ Department of Radiology, Feinberg School of Medicine Northwestern University Chicago Illinois USA; ^2^ Department of Biomedical Engineering Northwestern University Chicago Illinois USA; ^3^ Department of Medicine, Feinberg School of Medicine Northwestern University Chicago Illinois USA; ^4^ Department of Neurology, Feinberg School of Medicine Northwestern University Chicago Illinois USA

**Keywords:** arterial pulsatility, cerebral perforating arteries, cerebral small vessel disease (cSVD), dual‐VENC, phase‐contrast MRI, pulsatility index (PI)

## Abstract

**Background:**

Dysfunction of cerebral perforating arteries is a major contributor to cerebral small vessel disease. Developing a reliable MRI technique for assessing cerebral perforating arteries on widely accessible 3T systems would be advantageous.

**Purpose:**

To evaluate the feasibility and reliability of dual‐velocity encoding (dual‐VENC) PC‐MRI at 3T for assessing pulsatility of cerebral perforating arteries.

**Study Type:**

Prospective.

**Subjects:**

Twelve healthy young adults (2 female, 24.0 ± 3.99 years) and 31 older adults with and without vascular risk factors (21 female, 67.72 ± 8.48 years).

**Field Strength/Sequence:**

Dual‐VENC 2D PC‐MRI at 3T and 7T.

**Assessment:**

The number of perforators (N_perforator_) and pulsatility index (PI) measured using 3T dual‐VENC PC‐MRI were evaluated through test–retest and comparison against those by 7T dual‐VENC PC‐MRI on the younger participants. The associations of PI and N_perforator_ with age, cognition, and vascular risk factors were investigated in the elderly cohort.

**Statistical Tests:**

Paired *t*‐tests, two‐sample *t*‐tests, Bland–Altman analysis, coefficient of variation (CV), Shapiro–Wilk Test, one‐way ANOVA, and multivariable regression models. Significance level: 0.05.

**Results:**

3T dual‐VENC PC‐MRI provided better reproducibility with CV values of 10% and 14% for PI and N_perforator_, respectively, compared to single VENCs (high VENC: 21% and 21%, low VENC: 13% and 14%). 3T dual‐VENC PC‐MRI showed no significant difference in N_perforator_ and PI measurements with 7T dual‐VENC (*p* = 0.16, 0.38, respectively). Among the older participants, aging and cognitive impairment were both significantly associated with increased PI but not with N_perforator_ (*p* = 0.17 and 0.365); global vascular risk burden, as well as individual vascular risk factors, including pulse pressure and hypercholesterolemia, showed a significant association with PI but not with N_perforator_ (*p* = 0.858, 0.345, and 0.476).

**Data Conclusion:**

3T dual‐VENC PC‐MRI provides high‐fidelity pulsatility assessment of cerebral perforating arteries and may be a useful tool at widely accessible 3T.

**Level of Evidence:**

Level 2.

**Technical Efficacy:**

Stage 2.

## Plain Language Summary

Dysfunction of cerebral perforating arteries may be linked to cerebral small vessel disease. While 2D phase‐contrast MRI (PC‐MRI) at 7T has been successfully applied to assess flow pulsatility in these tiny arteries, 7T has not yet achieved widespread use. This study introduced a dual‐velocity‐encoding (dual‐VENC) PC‐MRI technique to assess cerebral perforating arteries at widely accessible 3T. 3T dual‐VENC provided good reproducibility and comparable pulsatility measurements to 7T. Our aging study showed that pulsatility index was associated with age and vascular risk factors. These results suggest that 3T dual‐VENC PC‐MRI can be a reliable imaging tool for assessing cerebral perforating arteries.

## 1 Introduction

Cerebral small vessel disease (cSVD) is a common, chronic, and progressive vascular disease in elderly adults, responsible for approximately 25% of strokes and at least 40% of dementia cases [1]. The development of imaging biomarkers is important for effective prevention and treatment of cSVD. Arteriolosclerosis is recognized as the major vascular pathology of cSVD, which refers to pathological changes of cerebral perforating arteries (e.g., lenticulostriate arteries [LSAs]) [2]. These small vessels play an important role in maintaining the metabolic activities of deep structures of the brain, such as the basal ganglia region, by supplying blood flow to the deep nuclei [3]. Dysfunction of perforating arteries can lead to lacunar infarcts, basal ganglia hemorrhages, and deep microbleeds [3, 4, 5]. Therefore, directly characterizing the dysfunction of cerebral perforating arteries could help provide useful insights into the cSVD pathology.

Arterial flow pulsatility quantified by the pulsatility index (PI) has been recognized as a useful indicator of vascular dysfunction and can be measured from blood flow velocity waveforms assessed by phase‐contrast MRI (PC‐MRI) [6, 7]. Previous studies have demonstrated that flow velocity and pulsatility of cerebral perforating arteries can be reliably characterized using higher‐resolution 2D PC‐MRI at ultra‐high field 7T [8, 9, 10]. Recent 7T studies have reported that a higher PI of cerebral perforating arteries is linked to cSVD [11, 12], indicating that arterial pulsatility of cerebral small arteries could serve as a sensitive marker of cSVD. However, 7T MRI is not yet in widespread clinical use due to its substantial cost, inherent technical challenges such as B1/B0 inhomogeneities, and high specific absorption rate (SAR) [13, 14]. To facilitate broader applicability in both clinical and research settings, development of a reliable imaging technique for assessing flow pulsatility of cerebral perforating arteries at widely accessible 3T MRI would be advantageous.

Recent studies from Arts et al. and van Tuijl et al. have shown the possibility of measuring flow pulsatility of cerebral perforating arteries, that is, LSAs, using conventional PC‐MRI at 3T [15, 16]. Although their findings were promising, both studies have limitations such as a relatively low signal‐to‐noise ratio (SNR), resulting in reduced sensitivity of perforator detection and discordance of PI measurements with those from 7T MRI. More recently, a dual‐velocity encoding (dual‐VENC) strategy has been introduced to improve the sensitivity and reliability of perforating artery detection and PI measurements with PC‐MRI at 7T [17]. Compared to the conventional single‐VENC approach, dual‐VENC improves the velocity to noise ratio (VNR) [18, 19, 20, 21] and thus increases the sensitivity of small vessel detection and flow velocity characterization, which may overcome the low SNR limitation of 3T. We hypothesized that 3T dual‐VENC PC‐MRI can provide high‐fidelity flow pulsatile assessment of cerebral perforating arteries.

Thus the aims of this study were to evaluate the feasibility, reliability, and sensitivity of characterizing flow pulsatility of cerebral perforating arteries using submillimeter‐resolution dual‐VENC PC‐MRI on widely available 3T systems through test–retest reproducibility, comparison with 7T, and investigating the effects of aging and vascular risk factors in older participants.

## 2 Materials and Methods

### 2.1 Study Participants

This study was approved by the local Institutional Review Board and all subjects provided written informed consent. Twelve healthy young volunteers (2 female, 24.08 ± 3.99 years) and thirty‐one older participants with or without vascular risk factors (21 female, 67.72 ± 8.48 years) took part in this study. All participants were scanned on a 3T MAGNETOM Prisma MRI system (Siemens Healthcare, Erlangen, Germany).

### 2.2 MRI Acquisition

A 3D high‐resolution time‐of‐flight (TOF) scan was performed to locate cerebral perforating arteries using the following parameters: field of view (FOV) = 158 × 200 × 75 mm^3^, voxel size = 0.2 × 0.2 × 0.4 mm^3^, repetition time (TR) = 12 ms, echo time (TE) = 4.67 ms, flip angle = 17°. Retrospectively gated single‐slice 2D phase‐contrast MRI (PC‐MRI) data were acquired in each participant. The imaging slice was placed perpendicular to most perforating arteries by taking the TOF angiogram as a reference (Figure S1). MRI data with VENCs of 20 cm/s (low) and 40 cm/s (high) were acquired with two sequential scans in each young participant and interleaved acquired within a single scan in each older participant. The acquisition schemes were illustrated in Figure S2. Foam padding was placed around the head to minimize motion during the scan.

### 2.3 Comparison Between Dual‐VENC and Single‐VENCs at 3T

We firstly compared the performance of the dual‐VENC method with conventional single‐VENC for characterizing flow pulsatility of cerebral perforating arteries at 3T. PI and the number of detected perforators (N_perforator_) measured by dual‐VENC were evaluated against those measured with the single‐VENCs of 20 and 40 cm/s in the 12 young volunteers using the imaging parameters listed in Table 1.

**TABLE 1:** Imaging parameters of dual‐VENC PC‐MRI at 3T and 7T.

Imaging parameters	3T	7T
FOV, mm (RL × AP)	180 × 180	180 × 178
Voxel size, mm	0.2 × 0.2 × 2	0.2 × 0.2 × 2
Flip angle °	60	58
VENC, cm/s	20/40	20/40
Bandwidth, Hz/pixel	130	130
TE, ms	11.84	9.72
Temporal resolution, ms	~100	95
N_average_	5	3
Scanning time, min	6	6

Abbreviations: AP, anterior–posterior; FOV, field of view; RL, right–left; VENC, velocity encoding.

### 2.4 Test–Retest Reproducibility of Dual‐VENC PC‐MRI at 3T

To assess the reproducibility of N_perforator_ and PI measurements in cerebral perforating arteries using dual‐VENC PC‐MRI at 3T, test & retest scans were performed in 10 out of the 12 young participants (1 female, 24.50 ± 4.28 years). High‐resolution PC‐MRI with dual‐VENC using identical imaging parameters as listed in Table 1 was repeated on each participant within the same imaging session.

### 2.5 Comparison Between 3T and 7T Dual‐VENC PC‐MRI

The reliable performance of 7T dual‐VENC PC‐MRI in characterizing flow pulsatility of cerebral perforating arteries has been demonstrated in a previous study [17]. To assess the reliability of dual‐VENC PC‐MRI at 3T, 9 of the 12 healthy participants (1 female, 24.22 ± 4.63 years) also underwent a dual‐VENC PC‐MRI scan on a Siemens Terra 7T MRI scanner (Siemens Healthcare, Erlangen, Germany) using an 8‐transmit/32‐receive head coil (Nova Medical, Wilmington, MA) with closely matched imaging position (Figure S1) and imaging parameters, as detailed in Table 1. Considering the inherent SNR difference between 3T and 7T and reasonable scan time, PC‐MRI with 5 averages was acquired at 3T while 3 averages were obtained for the 7T scans. To minimize environmental and physiological variability, participants were scanned on the same day for both 3T and 7T sessions.

### 2.6 Sensitivity Evaluation of 3T Dual‐VENC PC‐MRI With Aging

Previous studies have shown that aging is accompanied by increased PI of cerebral perforating arteries using 7T PC‐MRI [17, 22]. To evaluate the sensitivity of 3T dual‐VENC PC‐MRI in detecting age effects, the 31 older participants were imaged using dual‐VENC PC‐MRI at 3T. Instead of sequential PC‐MRI acquisitions of the two VENCs (as used in the younger study group), a single scan with interleaved VENC acquisition was performed on each older participant. This was more suitable for dual‐VENC data acquisition as it reduced the potential for head motion between two VENC acquisitions. The other imaging parameters closely matched those applied in the younger subjects, except that the scan time was slightly reduced, and true temporal resolution was 111 ms.

For each older participant, years of education, blood pressure, diabetes mellitus, cholesterol levels, and smoking history were recorded, and cognition was assessed with the Montreal Cognitive Assessment (MoCA) and Mini‐Mental State Examination (MMSE). Five vascular risk factors were considered: hypertension, hypercholesterolemia, diabetes mellitus, smoking history (defined as > 100 cigarettes in lifetime), and obesity (body mass index > 30 kg/m^2^). Consistent with prior studies [23], each factor was coded as a binary variable (1 = present, 0 = absent or remote), yielding a composite vascular risk score (cVRS) ranging from 0 to 5 to assess global vascular risk burden. The older participants were further separated into 3 subgroups, according to their cVRS scores (cVRS ≤ 1, cVRS = 2, cVRS = 3).

### 2.7 Image Processing

Postprocessing was conducted by JT (4 years of experience) using in‐house developed software written in MATLAB (version 2022b, MathWorks, Natick, MA). Both dual‐VENC and single‐VENC PC‐MRI images were processed following the steps published in a previous study [17], including (1) Generating the maximum intensity projection (MIP) from the PC‐MRI magnitude images across phases; As for dual‐VENC, MIPs were generated from the low VENC images; (2) Generating a perforator mask automatically using K‐means clustering [24] on the generated MIP image, followed by visual inspection, which largely reduced operator‐associated bias on the mask generation; (3) Calculating N_perforator_ using connected component analysis [25]; (4) For dual‐VENC, antialiasing was conducted on the low VENC phase maps using the high VENC phase maps on a pixel‐by‐pixel basis [26], according to Equation (1):

(1) $$V=V_{\mathrm{low}}+2V_{\mathrm{enc}-\mathrm{low}}k$$

where *k* is defined as the number of phase wraps. In contrast, antialiasing in single VENC data was empirically done in the blind of the other VENC data. (5) Extracting the velocity waveform from each detected LSA perforator in the mask on PC‐MRI phase maps. The perforators in which velocity curves displayed negative values (e.g., veins) or inconsistent waveforms (e.g., different peak cardiac cycles) were excluded from further analysis as they were most likely veins [27]; (6) Normalizing the velocities for each perforator by dividing the velocities by the mean velocity; and (7) PI was calculated according to Equation (2), and the mean PI was obtained by averaging the normalized velocities from all identified perforating arteries.

(2) $$\begin{array}{c} \mathrm{PI}=\frac{V_{\max}-V_{\min}}{V_{\text{mean}}} \end{array}$$

### 2.8 Statistical Analysis

All statistical analyses were conducted using R (v3.6.1, Austria) in R Studio (v1.2.5019, RStudio Inc., USA). The Shapiro–Wilk test was first applied to assess the normality of PI and N_perforator_. Depending on data distribution, either a paired *t*‐test or a Wilcoxon rank‐sum test was used to compare N_perforator_ and PI values between 3T and 7T. The agreements between 3T and 7T measurements were evaluated using Bland‐Alterman plots. The one‐way ANOVA or Kruskal–Wallis *H* test was used to compare PI among single VENCs of 20 and 40 cm/s, and dual‐VENC and three cVRS groups. Bland–Altman analysis and CV values were used to assess the test–retest reproducibility of N_perforator_ and PI measurements. To assess the associations of N_perforator_ and PI with individual vascular risk factors, multivariable linear regression analyses were performed using N_perforator_ and PI as dependent variables and vascular risk factors (systolic and diastolic blood pressure, hypercholesterolemia, diabetes mellitus, and smoking status) as independent variables. To evaluate the relationship of PI and N_perforator_ with cognitive function, multiple linear regression models were used including PI and N_perforator_ as the independent variables and continuous cognitive scores as the dependent variable. Results were reported as odds ratios (ORs) with corresponding 95% confidence intervals (CIs). All vascular risk models were adjusted for covariates, including age, sex, and years of education, where applicable. Statistical significance was defined as a two‐sided *p* value < 0.05.

## 3 Results

### 3.1 Comparison Between Dual‐VENC and Single‐VENC at 3T

**FIGURE 1:**
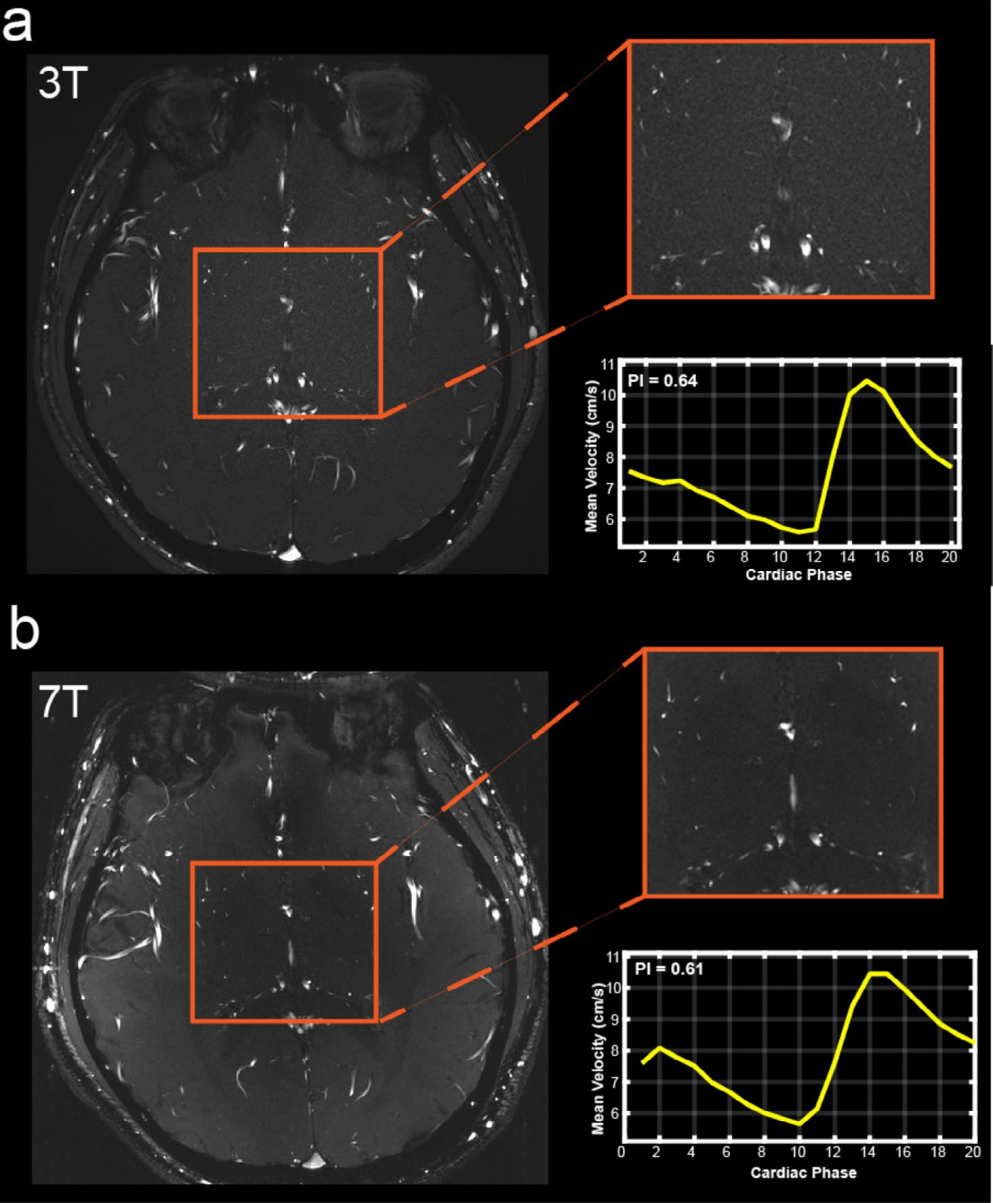
Low VENC (20 cm/s) magnitude images from a dual‐VENC PC‐MRI acquisition with zoomed‐in view showing detected perforators as well as the extracted velocity waveform at both 3T (a) and 7T (b) from the same representative case. The image contrast was adjusted using the window center and window width acquired from the DICOM information for better and direct visual assessment.

**FIGURE 2:**
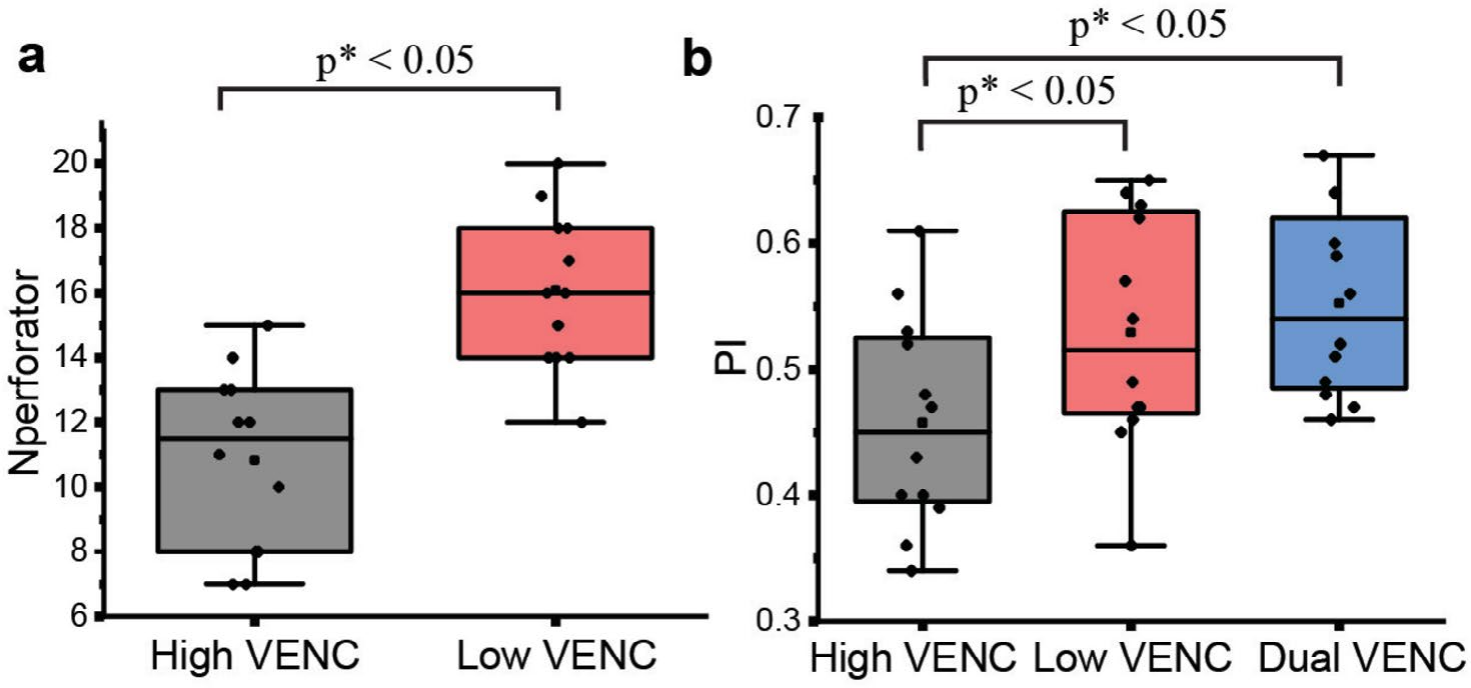
Comparison of the number of detected perforators (N_perforator_) (a) and PI measurements (b) using single low VENC (20 cm/s), single high VENC (40 cm/s), and dual‐VENC PC‐MRI at 3T in 11 young healthy subjects.

Cerebral perforating arteries were successfully detected by dual‐VENC PC‐MRI at 3T in all participants. Figure 1a shows an example case of PC‐MRI images with VENC of 20 cm/s at 3T, showing the successful detection of cerebral perforators, together with the pulsatile flow waveform across the cardiac cycle which was extracted from the detected perforators. Figure 2 shows the group comparison in N_perforator_ between the low and high VENCs. Significantly more perforators were detected with the lower VENC of 20 cm/s (N_perforator_ = 14.2 ± 2.1) compared to the 40 cm/s VENC (N_perforator_ = 8.1 ± 2.4). The average PI values across subjects were 0.55 ± 0.07, 0.49 ± 0.14, 0.53 ± 0.09 for the dual‐VENC and single VENCs of 40 and 20 cm/s, respectively. PI values with high‐VENC were significantly lower compared to those measured by both low VENC and dual‐VENC. While there was no difference in PI values between low‐VENC and dual‐VENC (*p* = 0.50), dual‐VENC showed reduced variations across subjects in PI measurements.

### 3.2 Test–Retest Reproducibility of Dual‐VENC PC‐MRI at 3T

**FIGURE 3:**
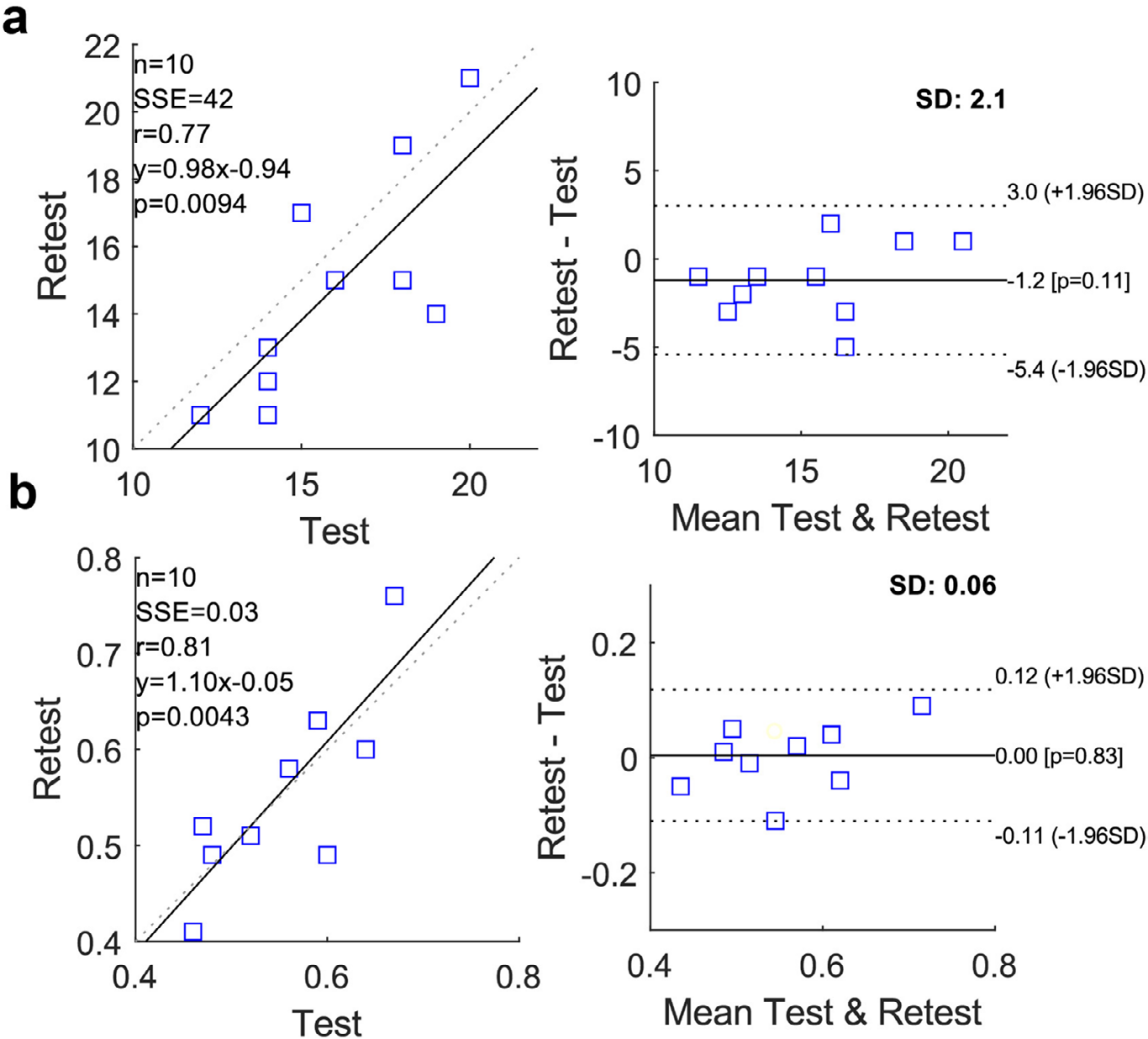
Linear correlations and Bland–Altman plots of test–retest measurements of N_perforator_ (a) and PI (b) using 3T dual‐VENC PC‐MRI in 10 young healthy subjects.

Figure 3a,b show linear correlation and Bland–Altman plots of N_perforator_ and PI measurements by dual‐VENC PC‐MRI at 3T, respectively. Strong correlations were observed for both PI (*r* = 0.81) and N_perforator_ (*r* = 0.77). The Bland–Altman plots showed the limits of agreement (LoA) of N_perforator_ and PI were [−5.4, 3.0] and [−0.11, 0.12], respectively, and the average standard deviations of the differences between tests were 2.1 and 0.06, respectively. Figures S3 and S4 present linear correlations and Bland–Altman plots of N_perforator_ and PI measurements from single VENC analyses of 20 cm/s and 40 cm/s, respectively. Between the two single VENCs, the lower VENC of 20 cm/s showed a higher correlation coefficient in both PI and N_perforator_ (*r* = 0.71 and 0.77) compared to the VENC of 40 cm/s (*r* = 0.4, 0.28). Nevertheless, compared to dual‐VENC, both single‐VENCs showed poorer reproducibility with reduced correlation coefficients and greater test–retest variations in PI measurements (*r* = 0.71 and 0.28; std. = 0.07 and 0.09 for VENCs of 20 and 40 cm/s, respectively). Consistently, the dual‐VENC PI showed lower CV compared to both single VENCs of 20 and 40 cm/s (10%, 13%, and 21%, respectively), and the N_perforator_ with low VENC showed lower CV compared to high‐VENC (14% and 21%, respectively).

### 3.3 Comparison of Dual‐VENC PC‐MRI Between 3T and 7T

**FIGURE 4:**
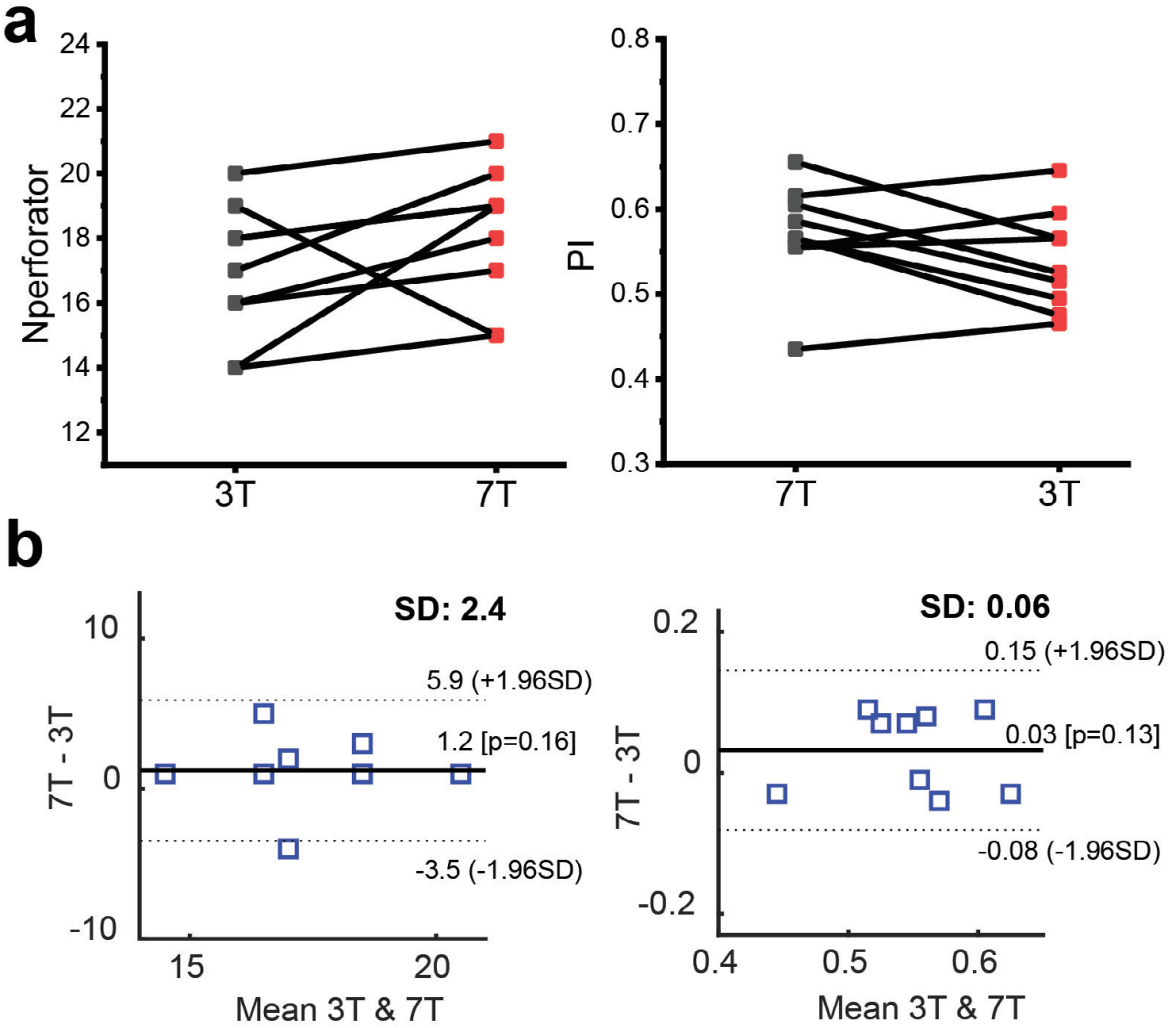
Comparison of the number of detected perforators (N_perforator_) (a) and pulsatility index (PI) (b) across subjects using dual‐VENC PC‐MRI at 3T and 7T.

Cerebral perforating arteries and their velocity waveforms across a cardiac cycle were successfully depicted using dual‐VENC PC‐MRI at 7T. Figure 1b shows an example 7T PC‐MRI image as well as the extracted mean flow waveform of the detected perforators from the same representative case as in Figure 1a. Closely matched delineations of perforators and their flow waveforms between 3T and 7T were observed, although 3T PC‐MRI magnitude images looked slightly noisier. Figure 4a,b compare the N_perforator_ and PI between 3T and 7T across the 9 healthy young subjects. For 3T and 7T, the average N_perforator_ was 16.9 ± 2.1 and 18.1 ± 2.1, and the average PI was 0.54 ± 0.07 and 0.57 ± 0.06, respectively. No significant differences were detected in both N_perforator_ and PI measured by dual‐VENC PC‐MRI between 3T and 7T (*p* = 0.16, 0.38, respectively). The Bland–Altman plots (Figure 4c) demonstrated good agreement for N_perforator_ and PI measurements between 3T and 7T with a mean bias of 1.2 and 0.03, limits of agreement of [5.9, 3.5] and [0.15, −0.08], respectively.

### 3.4 Dual‐VENC PC‐MRI Measurements at 3T With Aging and Vascular Risk Factors

**FIGURE 5:**
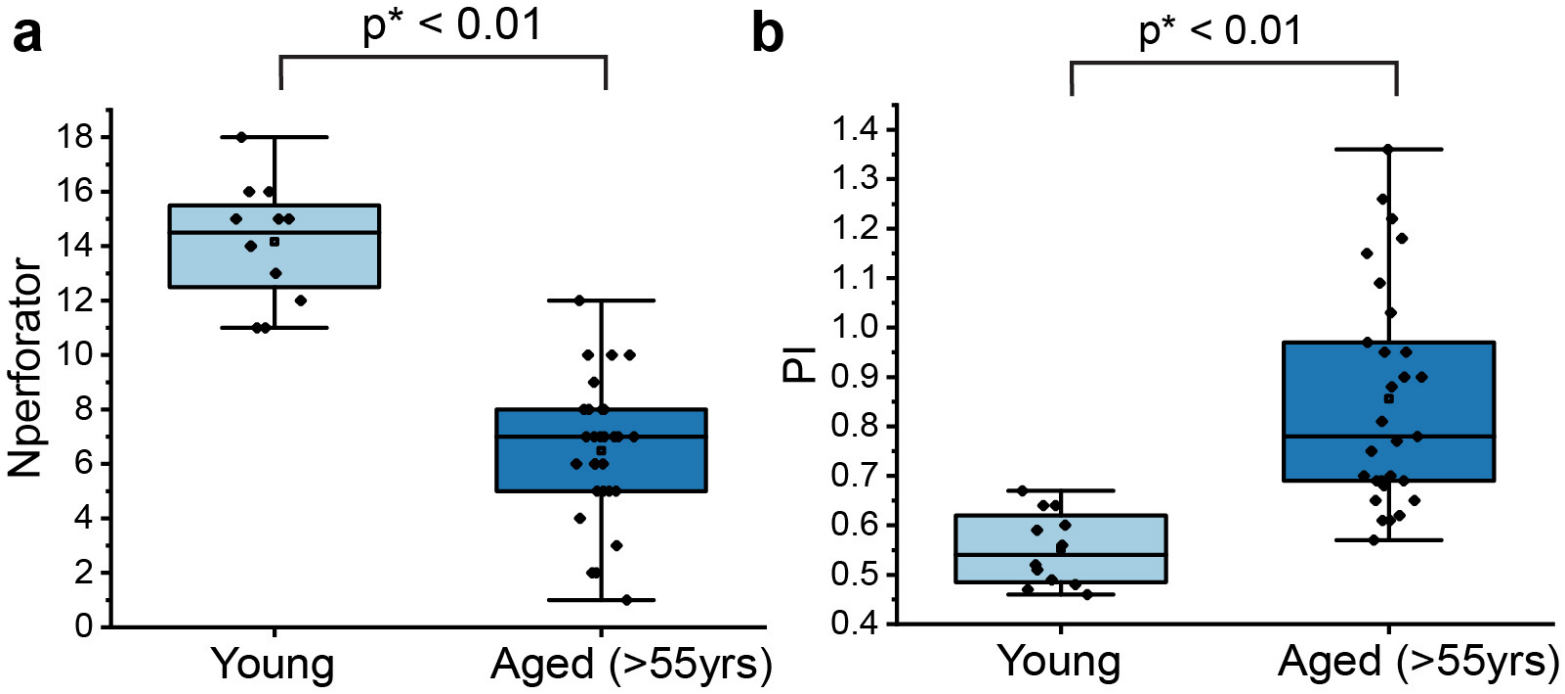
Boxplots of N_perforator_ (a) and PI (b) between younger and older groups.

**FIGURE 6:**
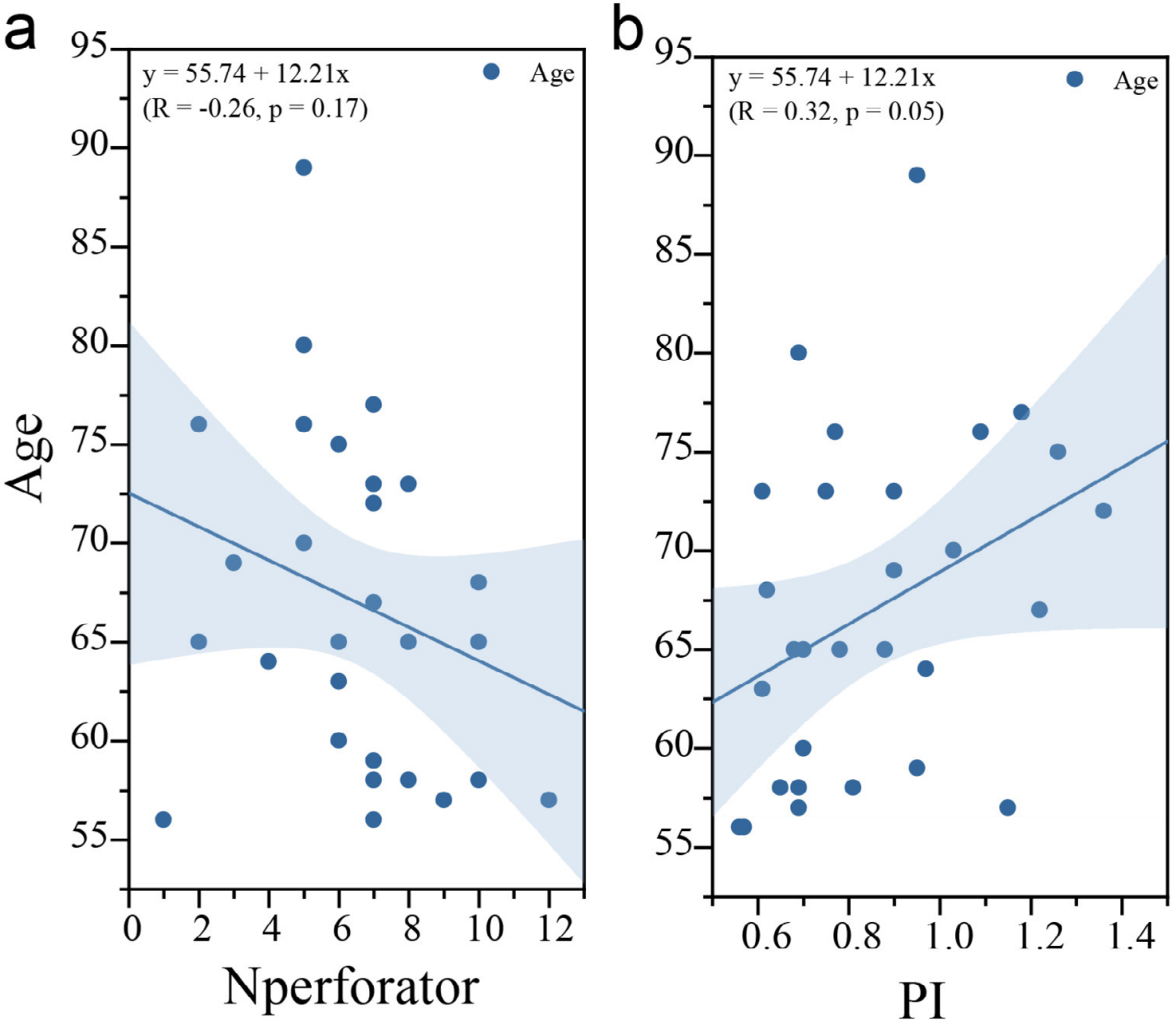
Scatter plots of number of perforators (N_perforator_) (a) and pulsatility index (PI) (b) with age.

Table 2 lists demographic information of the older participants including age, sex, results of cognitive tests, and vascular risk factors. Figure 5 shows the group comparison of dual‐VENC PC‐MRI measurements between younger and older participants. Significantly reduced N_perforator_ was observed in the older (6.7 ± 2.4) participants compared to that of the younger participants (14.2 ± 2.1) (Figure 5a). The mean PI value was also significantly higher in the older group (0.86 ± 0.22) compared to the younger group (0.55 ± 0.07) (Figure 5b). Among the older participants, PI increased significantly with age, while N_perforator_ was not significantly correlated with age (*p* = 0.17) (Figure 6). No significant sex‐dependent effects were observed in either PI or N_perforator_ (*p* = 0.28 and 0.064, respectively).

**TABLE 2:** Demographics of the older participants in this study.

Characteristic	Total (*n* = 31)
Age (years), mean ± SD	67.7 ± 8.5
Sex, *n* (%)	
Male	11 (35.5)
Female	20 (64.5)
Education, mean ± SD	26.1 ± 3.4
Comorbidities, *n* (%)	
Hypertension	13 (41.9)
Diabetes	3 (9.68)
Smoking	13 (41.9)
Hyperlipidemia	19 (61.3)
Cognitive scores, mean ± SD	
MoCA	25.6 ± 3.3
MMSE	28.6 ± 2.4
cVRS, *n* (%)	
≤ 1	17 (54.8)
= 2	9 (29.0)
≥ 3	5 (16.1)
LSA PI, mean ± SD	0.86 ± 0.22

Abbreviations: cVRS, vascular composite scores; LSA, lenticulostriate artery; MMSE, Mini‐Mental Status Exam; MoCA, Montreal Cognitive Test.

**FIGURE 7:**
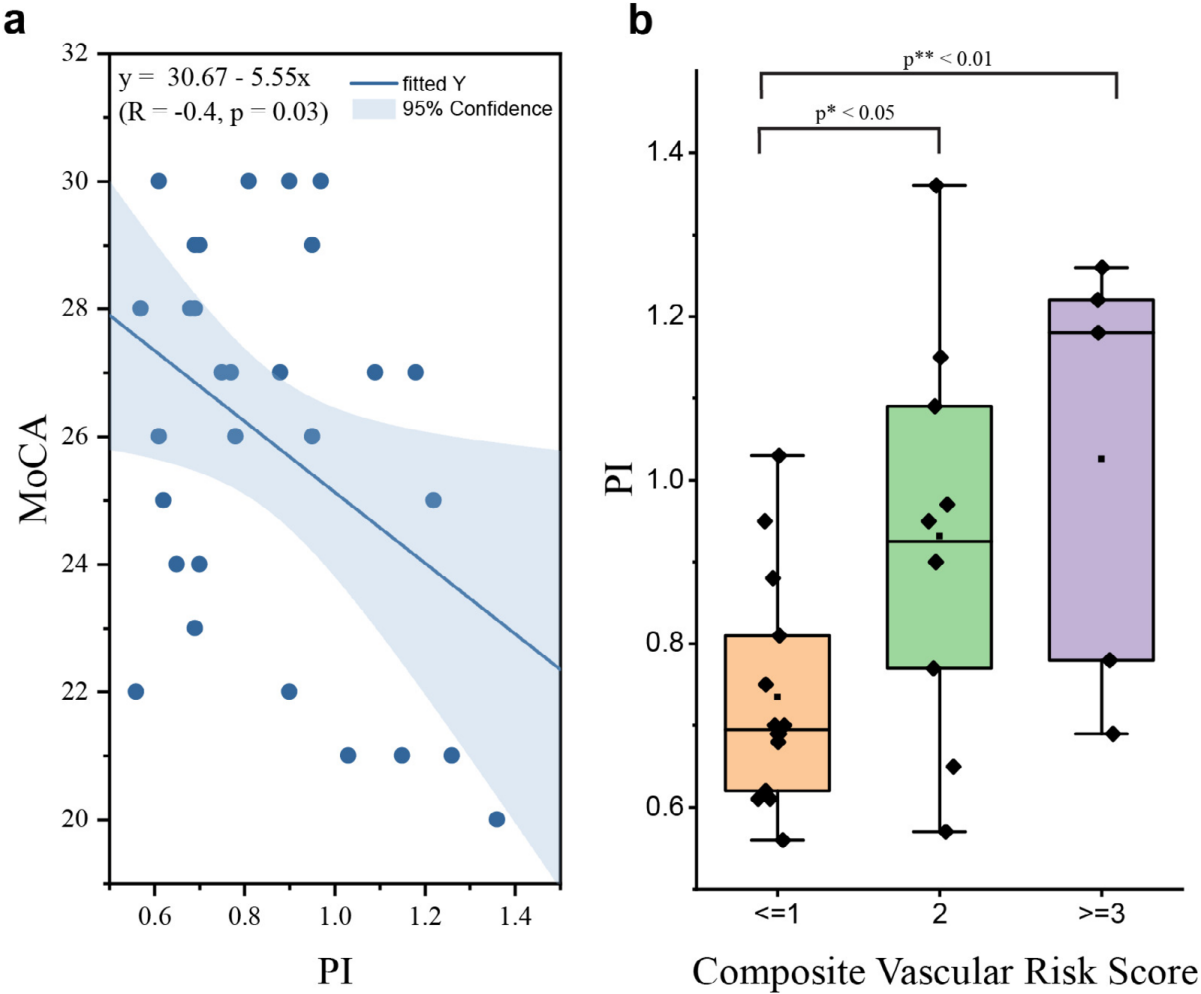
Scatter plot between pulsatility index (PI) values and MoCA scores (a) and boxplots of PI measurements and composite vascular risk scores (b).

In the older participants, higher PI values were associated with worse cognitive performance as measured by MoCA scores(Figure 7a), while no significant associations were found between N_perforator_ and MoCA (*p* = 0.365). No significant correlations were found between both PI, N_perforator_, and MMSE (*p* = 0.761, 0.114). A significant increase in PI was observed in elderly individuals with cVRS ≥ 2, compared to those with cVRS < 2 (Figure 7b). Higher PI was significantly associated with higher pulse pressure and the presence of hypercholesterolemia (Table 3), while no significant associations were observed between PI and systolic blood pressure, smoking history, or diabetes mellitus (*p* = 0.153, 0.242, 0.732). None of the vascular risk factors showed a significant relationship with N_perforator_ (*p* = 0.313, 0.345, 0.847, 0.476, 0.564).

**TABLE 3:** Regression coefficients from multivariable analysis: blood pressure, smoking history, hypercholesterolemia, and diabetes.

Outcome variable^a^	Predictor variable														
	Systolic pressure			Pulse pressure			Smoking			Hypercholesterolemia			Diabetes		
	*B*	95% CI	*p*	B	95% CI	*p*	*B*	95% CI	*p*	*B*	95% CI	*p*	*B*	95% CI	*p*
PI	0.003	[−0.001, 0.007]	0.153	0.006	[0.001, 0.012]	0.027	−0.095	[−0.259, 0.069]	0.242	0.181	[0.031, 0.331]	0.021	0.058	[−0.289, 0.405]	0.732
N_perforator_	0.022	[−0.023, 0.067]	0.313	0.029	[−0.034, 0.093]	0.345	0.190	[−1.821, 2.202]	0.847	0.643	[−1.194, 2.479]	0.476	−1.168	[−5.290, 2.953]	0.564

Abbreviations: *B*, beta coefficient; CI, confidence interval; N_perforator_, number of detected perforators; *p*, *p* value; PI, pulsatility index.

^a^ Adjusted for age, gender, and education.

## 4 Discussion

This study demonstrated the feasibility and reliability of characterizing flow pulsatility of cerebral perforating arteries using higher‐resolution dual‐VENC PC‐MRI at widely accessible 3T. The feasibility and reliability of dual‐VENC PC‐MRI at 3T were demonstrated through the test–retest reproducibility study and comparison with 7T. Compared to conventional single‐VENC PC‐MRI, dual‐VENC PC‐MRI showed better reproducibility in the PI assessment of cerebral perforating arteries at 3T. No significant difference in both perforator detection and PI measurements was obtained between 3T and 7T dual‐VENC PC‐MRI. Therefore, 3T dual‐VENC PC‐MRI has the potential to provide high‐fidelity pulsatility assessment of cerebral perforating arteries. The sensitivity of 3T dual‐VENC PC‐MRI as an imaging tool in studying aging was also evaluated in an aged cohort. Increased PI was significantly associated with aging, cognitive impairment, and global vascular risk burden, as well as individual vascular risk factors, including pulse pressure and hypercholesterolemia, though no significances were found with N_perforator_. These findings indicate that dual‐VENC PC‐MRI may be a promising technique to assess pulsatile flow dysfunction of cerebral perforating arteries in older adults using widely accessible 3T MRI. These findings are clinically important, as the development of reliable and sensitive imaging biomarkers of cerebral small vessels on widely accessible 3T magnets may facilitate wider applicability in clinical use.

In previous studies, the morphology and hemodynamics of cerebral perforating arteries have been primarily assessed using ultra‐high field 7T higher‐resolution MRI techniques due to their small vessel size [8, 27]. Reliably characterizing these small arteries on conventional field strength 3T scanners is challenging due to lower SNR. Previous studies have explored the possibility of imaging pulsatility using 2D PC‐MRI with a single VENC of 20 cm/s at 3T in reference to 7T [15, 16]. However, discordance between 3T and 7T MRI in both N_perforator_ and PI determinations was observed [15], which was likely attributed to the reduced inherent SNR at 3T and unreliable antialiasing correction with a single VENC. The size of cerebral perforating arteries varies from 0.1 to 1.28 mm [28]. The velocities within these vessels and across cardiac phases may change dramatically. Therefore, dual‐VENC may be an ideal technique to image cerebral perforating arteries, as it can offer better VNR and thus improve the depiction of flow velocity dynamics, especially in vessels with a broader velocity range, compared to single‐VENC [18]. In this study, PI values measured by 3T dual‐VENC PC‐MRI were in agreement with those reported in previous studies [8, 10]. Consistent with previous 7T findings [17], low VENC detected significantly more perforators than high VENC, and PI measured by dual‐VENC showed reduced variations across subjects compared to single VENC. In this study, PI with high VENC was significantly lower than both low VENC and dual‐VENC, which is speculated to be caused by reduced sensitivity to characterize some smaller perforating arteries with the high VENC. However, given the large variations possessed in the PI values measured by both single VENCs, the interpretation of this finding needs to be cautious, and further evaluation with a large sample size is needed. Dual‐VENC showed improved test–retest reproducibility in PI measurement, compared to single VENC. With dual‐VENC, the detection of perforating arteries and PI measurements at 3T has the potential to achieve performance comparable to that at 7T, providing the opportunity to study hemodynamic dysfunction of cerebral perforating arteries using conventional 3T instead of using high‐cost and limited clinical accessibility of 7T.

Our findings show age‐related PI increases using 3T dual‐VENC PC‐MRI. This observation is consistent with prior studies [11, 22, 29, 30] that showed both larger and smaller cerebral arteries and suggest that aging is accompanied by arterial stiffening and increased arterial pulsatility throughout the cerebral vasculature. Contrary to a previous 7T study [17], in this study, 3T dual‐VENC PC‐MRI detected a significant reduction in N_perforator_ in the older participants compared to the young participants. This discrepancy may be explained by the challenges of detecting smaller perforators with much slower flow velocities on 3T, given its inherent lower SNR. Another possible explanation is that this study employed automatic perforator detection using K‐means clustering instead of manual selection which was used in the previous study [17], potentially improving the accuracy and reliability in perforator detection.

Vascular risk factors, such as hypertension, diabetes mellitus, high cholesterol, and smoking, could lead to stroke, cognitive impairment, and dementia by causing cerebral small vessel dysfunction or permanent vessel wall damage [31]. In our study, a higher PI of cerebral perforating arteries was associated with increased global vascular risk burden and higher individual vascular risk factors, such as pulse pressure and hyperlipidemia. However, no significant correlation was found between PI and either diabetes mellitus or smoking status, consistent with a previous study [32]. Older adults with higher PI values showed significantly poorer cognitive performance on the MoCA. Interestingly, N_perforator_ was not significantly associated with age, vascular risk factors, and cognition. This may be in part explained by the fact that the majority of the older adults participating in this study were cognitively normal or had only mild cognitive impairment and had not yet reached a threshold of dysfunction or damage to the cerebral small vessels. Alternatively, it may suggest that hemodynamic dysfunction, such as increased PI, is more closely linked with vascular risk factors and cognitive impairment in aged individuals. Thus, the findings from our older participant group highlight the potential of 3T dual‐VENC PC‐MRI as a promising tool to assess early vascular dysfunction or damage of cerebral perforating arteries. Although these findings are encouraging, the sensitivity and reliability of 3T dual‐VENC in characterizing cerebral perforating arteries need to be further evaluated when applied to patients with cSVD and mild cognitive impairment, particularly those with regional infarction and white matter hyperintensities who might present narrowed vessels and dramatically reduced flow velocities.

## 5 Limitations

First, the 3T versus 7T comparison was conducted only in a small cohort of healthy young adults. A comprehensive evaluation with a larger sample size and a more diverse population according to age and race/ethnic group is needed for a robust evaluation. Secondly, two sequential single‐VENC acquisitions instead of a single interleaved dual‐VENC acquisition were acquired in all younger participants, which may be susceptible to inter‐scan motion. To mitigate it, in this study, foam padding was carefully placed around the head to minimize motion; we also thoroughly examined the data before post‐processing. No evident motions were found between the two VENC datasets in the young subjects. Thirdly, our dual‐VENC approach currently relies on basic phase unwrapping algorithms. Incorporating more advanced denoising and phase correction techniques could further improve the accuracy of velocity and pulsatility measurements [18, 33, 34]. Fourthly, the current scan time ranged from 6 to 8 min. Future work could explore the use of different k‐t acceleration strategies to further improve the acquisition efficiency [19, 35]. Finally, our aging study focused primarily on the relationship between PI of perforating arteries with aging, vascular risk factors, and cognitive measures. As indicated by previous studies that increased PI of cerebral perforating arteries may contribute to cSVD pathology [11, 12], future research will be conducted to investigate the relationship between flow pulsatility of cerebral perforating arteries with established cSVD neuroimaging makers, such as enlarged perivascular spaces and white matter hyperintensities, as well as the damping factor, which may offer further insight into the role of dysfunction of cerebral perforating arteries in cSVD [36, 37, 38].

## 6 Conclusion

3T dual‐VENC 2D phase‐contrast MRI can be a feasible and reliable method for characterizing flow pulsatility in cerebral perforating arteries, supported by good test–retest reproducibility and agreement with 7T dual‐VENC PC‐MRI. PI of cerebral perforating arteries showed significant associations with age, cognition, and vascular risk factors, while no significant relationship was found with the number of detected perforators. These findings suggest that 3T dual‐VENC PC‐MRI could offer a promising and accessible approach for studying vascular dysfunction in cerebral perforating arteries.

## Funding

National Institute of Health (NIH) grants R01NS118019, RF1AG072490, RF1NS139370, and BrightFocus Foundation A20201411S.

## Supporting information


**Figure S1:** Time of flight (TOF) images acquired at 3T and 7T with a yellow line indicating the imaging position of dual‐VENC PC‐MRI.
**Figure S2:** Illustration of the PC‐MRI acquisition schemes including (a) sequential dual‐VENC acquisition consisting of two single‐VENC scans and (b) interleaved dual‐VENC acquisition within a single scan.
**Figure S3:** Bland–Altman plots of test–retest measurements of Nperforator (a) and PI (b) with a single VENC = 20 cm/s.
**Figure S4:** Bland–Altman plots of test–retest measurements of Nperforator (a) and PI (b) with the single VENC = 40 cm/s.
