# Supplementary material for: Pulsatility Assessment of Cerebral Perforating Arteries Using Submillimeter‐Resolution Dual‐VENC Phase‐Contrast MRI at 3T
Source: J Magn Reson Imaging. 2025 Dec 27;63(5):1246–56. doi: 10.1002/jmri.70218 (PMC12990146; doi:10.1002/jmri.70218)
Supplement: Supplementary file 1 — Figure S1: Time of flight (TOF) images acquired at 3T and 7T with a yellow line indicating the imaging position of dual‐VENC PC‐MRI. Figure S2: Illustration of the PC‐MRI acquisition schemes including (a) sequential dual‐VENC acquisition consisting of two single‐VENC scans and (b) interleaved dual‐VENC acquisition within a single scan. Figure S3: Bland–Altman plots of test–retest measurements of Nperforator (a) and PI (b) with a single VENC = 20 cm/s. Figure S4: Bland–Altman plots of test–retest measurements of Nperforator (a) and PI (b) with the single VENC = 40 cm/s. [file JMRI-63-1246-s001.docx]

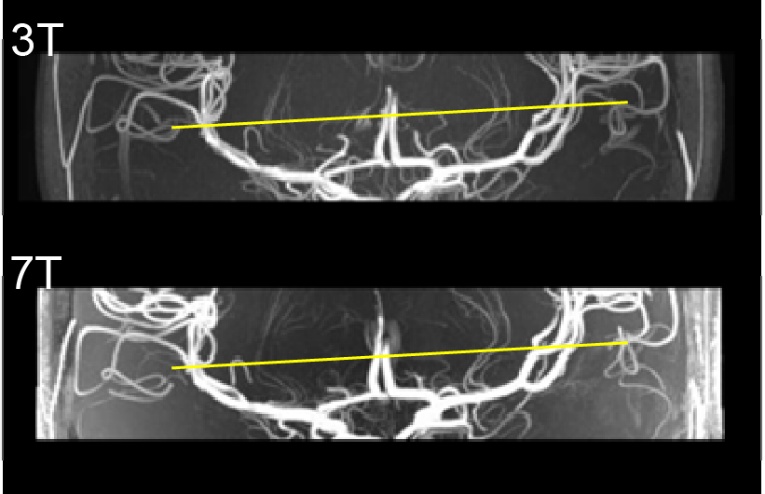


Supplementary Figure S1. Time of Flight (TOF) images acquired at 3T and 7T with a yellow line indicating the imaging position of dual-VENC PC-MRI.


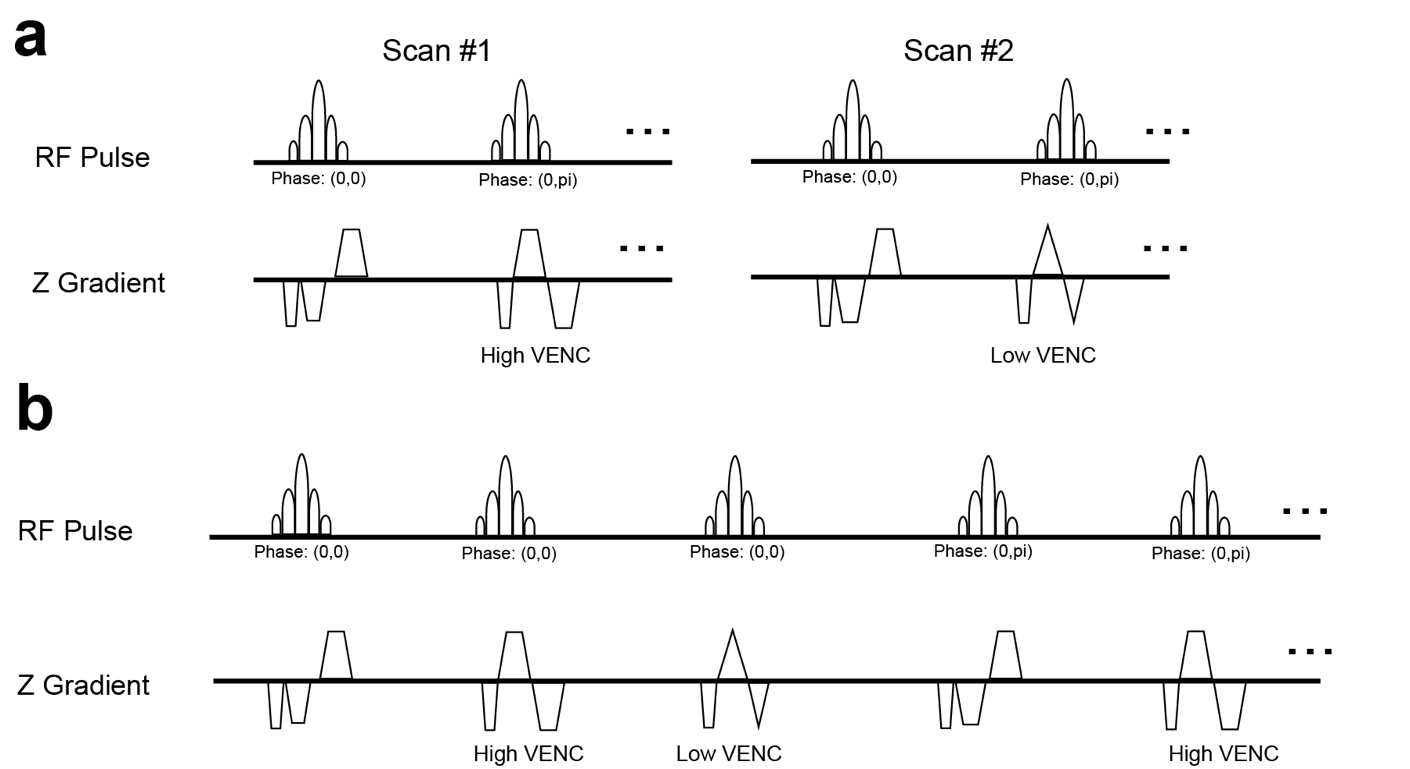


Supplementary Figure S2. Illustration of the PC-MRI acquisition schemes including (a) sequential dual-VENC acquisition consisting of two single-VENC scans and (b) interleaved dual-VENC acquisition within a single scan.


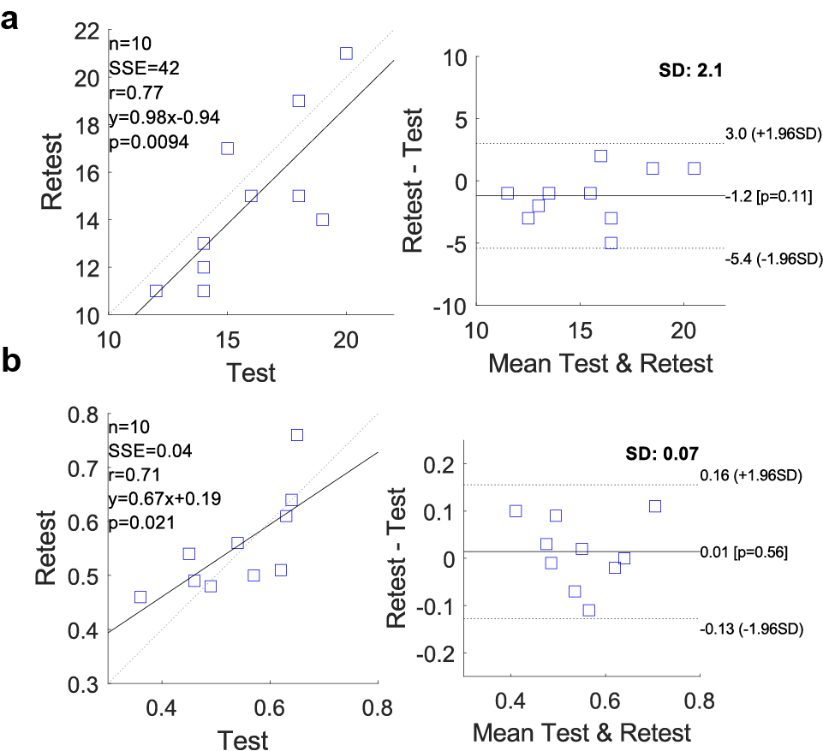


Supplementary Figure S3. Bland–Altman plots of test–retest measurements of N_perforator_ (a) and PI(b) with a single VENC = 20cm/s.


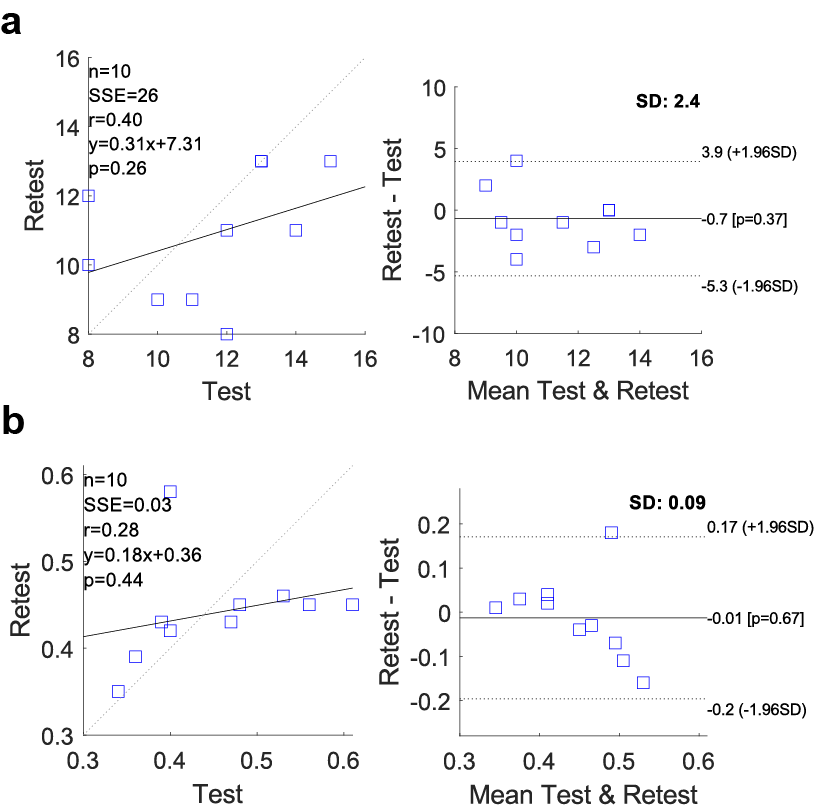


Supplementary Figure S4. Bland–Altman plots of test–retest measurements of N_perforator_ (a) and PI(b) with the single VENC = 40cm/s.
